# Positionally distinct interferon-stimulated dermal immune-acting fibroblasts promote neutrophil recruitment in Sweet syndrome

**DOI:** 10.1016/j.jaci.2025.05.029

**Published:** 2025-06-16

**Authors:** Kellen J. Cavagnero, Julie Albright, Fengwu Li, Edward Liu, Tatsuya Dokoshi, Rachael Bogle, Joseph Kirma, J. Michelle Kahlenberg, Allison C. Billi, Jennifer Fox, May P. Chan, Anthony Coon, Craig J. Dobry, Brian Hinds, Lam C. Tsoi, Paul W. Harms, Johann E. Gudjonsson, Richard L. Gallo

**Affiliations:** a Department of Dermatology, University of California San Diego, La Jolla; b Department of Dermatology, University of Michigan, Ann Arbor; c Division of Rheumatology, Department of Internal Medicine, University of Michigan, Ann Arbor; d Taubman Medical Research Institute, Ann Arbor; e Department of Pathology, University of Michigan, Ann Arbor

**Keywords:** Neutrophilic dermatoses, Sweet syndrome, inflammation, fibroblast, interferon, neutrophil

## Abstract

**Background:**

Sweet syndrome is an inflammatory skin disease characterized by robust neutrophil infiltration into the dermis. The pathogenesis of Sweet syndrome and its distinguishing features compared to other neutrophilic dermatoses, such as pyoderma gangrenosum, remain poorly understood.

**Objective:**

Our aim was to define the cellular and molecular landscape of the skin of patients with Sweet syndrome.

**Methods:**

Single-nucleus and bulk transcriptomics were performed on archival clinical skin samples from patients with Sweet syndrome, patients with pyoderma gangrenosum, and healthy controls. For mechanistic validation, functional experiments were performed with primary human cells. Spatial transcriptomics with single-molecule resolution was used to map cell types to tissue location.

**Results:**

A prominent interferon signature was identified in Sweet syndrome skin that was reduced in tissue samples from patients with pyoderma gangrenosum and healthy controls. This signature was observed in different subsets of cells, including fibroblasts that expressed interferon-induced genes. Functionally, this response was supported by analysis of cultured dermal fibroblasts that were observed to highly express neutrophil chemokines in response to activation by type I interferon. Furthermore, spatial transcriptomics revealed 2 positionally distinct interferon-activated fibroblast subsets: CXCL1-positive fibroblasts near neutrophil infiltrates and CXCL12-positive fibroblasts distal to these infiltrates.

**Conclusion:**

This study defines the cellular and molecular landscape of neutrophilic dermatoses and implicates dermal immune-acting fibroblasts in Sweet syndrome pathogenesis through type I interferon recognition and neutrophil recruitment.

## Capsule summary

Single-nucleus, spatial, and bulk transcriptomics of neutrophilic dermatoses and healthy control skin identify a strong interferon-induced neutrophil chemokine response in Sweet syndrome dermal fibroblasts, implicating interferon and immune-acting fibroblasts specifically in Sweet syndrome pathogenesis.

## GRAPHICAL ABSTRACT

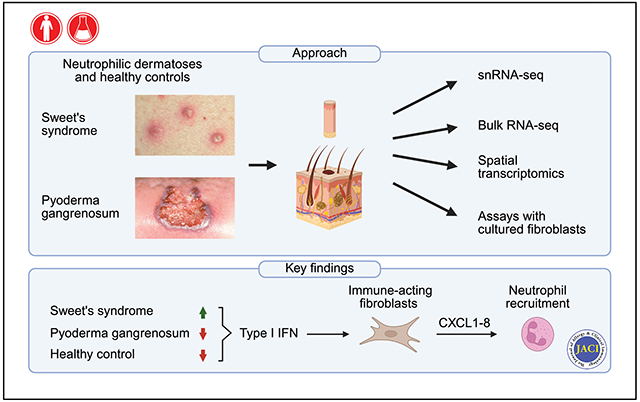

Sweet syndrome (SS) is an uncommon inflammatory skin disease characterized by painful red nodules or plaques with dense neutrophil infiltrate on the face, neck, or arms.^1^ Therefore, SS belongs to the class of neutrophilic dermatoses that also includes pyoderma gangrenosum (PG), pustular psoriasis, and Behçet disease. Although SS is often idiopathic, it can be drug induced and has been associated with various immune-related conditions such as cancer, infection, inflammatory diseases, vaccination, and pregnancy. The etiology of SS remains largely unknown but is thought to involve aberrant neutrophils^2^; genetic factors^2^; and proinflammatory cytokines such as IL-1, TNF-α, and IL-6.^1–3^ Although systemic steroids effectively treat many patients with SS, there is a pressing need for novel therapeutic approaches to address steroid resistance and minimize side effects.

Recent technologic advancements have enabled detailed transcriptomic analysis of fresh and formalin-fixed, paraffin-embedded (FFPE) tissue samples, providing an opportunity to comprehensively characterize gene expression at the single-cell and spatial levels during disease.^4^ Such analysis has advanced understanding of the pathophysiology of many diseases ranging from atopic dermatitis^5^ to inflammatory bowel disease^6^ and Alzheimer disease.^7^ Because of the difficulty of obtaining fresh tissue from patients with SS, a detailed characterization of gene expression in patients with SS has not been reported.

Here, we define the cellular and molecular landscape of skin from patients with SS, patients with PG, and healthy controls (HCs) using cutting-edge single-nucleus RNA sequencing (snRNA-Seq), bulk RNA sequencing (RNA-Seq), and subcellular resolution spatial transcriptomics approaches. Integrated analysis identified a prominent and unique type I interferon signature in patients with SS in various subsets of cells, including fibroblasts. Functional experiments with primary human dermal fibroblasts demonstrated that type I interferon activates these cells to highly express inflammatory mediators relevant to SS, including neutrophil chemokines. Overall, this study reveals a hitherto unknown role for type I interferon, through activation of immune-acting fibroblasts (IAFs), to promote neutrophil inflammation in SS.

## METHODS

Sex was not considered as a biologic variable. Biopsy samples were obtained in a deidentified manner from diagnostic cases and therefore lack associated clinical data, including sex. Whether the findings reported depend on sex is unknown.

### snRNA-Seq

FFPE skin biopsy samples were obtained from patients with SS (n = 6), patients with PG (n = 6), and HCs (n = 5). Libraries were generated using the 10X Genomics Flex FFPE protocol (10X Genomics, Pleasanton, Calif) and subjected to 28 × 91 bp of sequencing according to the manufacturer’s protocol (Illumina NovaSeq [Illumina, San Diego, Calif]). Library preparation and next-generation sequencing were carried out in the Advanced Genomics Core at the University of Michigan. CellRanger with default parameters was used to perform alignment to the hg38 reference genome and gene counting. Data were filtered, processed, and analyzed using Seurat.^8,9^ All functions described here are Seurat functions unless stated otherwise. Filtering data involved removing low-quality cells, removing ambient RNA with SoupX with setContaminationFraction = 0.2,^10^ and removing doublets using DoubletFinder with the default settings.^11^ Data were normalized and integrated using Normalize-Data and IntegrateData with default parameters. Clusters were identified using FindNeighbors with 50 principal components and FindClusters with a range of resolutions. For each resolution, nonlinear dimensionality reduction and visualization was performed with 50 principal components, and marker genes for each cluster were determined using FindAllMarkers with min. pct = 0.25. The resolution yielding clusters with the most distinct marker genes was chosen for further analysis. For cell subpopulation analysis, data were analyzed by subset based on cell type annotation, contaminating cells were removed, and the aforesaid analysis was repeated. Pathway analysis was performed with Metascape.^12^ Signature scores were generated using AddModule-Score with all significantly upregulated genes (adjusted *P* value < .05) from *in vitro* bulk RNA-Seq.

### Spatial transcriptomics

FFPE skin biopsy samples were obtained from patients with SS (n = 5) and HCs (n = 2). Subcellular resolution spatial transcriptomics was performed using the NanoString CosMx SMI platform with the predefined human 1000 gene panel as previously described.^13^ 4′,6-Diamino-2-phenylindole staining and immuno-fluorescent staining of pan-cytokeratin, CD298/B2M, CD45, and smooth muscle actin facilitated cell segmentation with machine learning algorithm Cellpose.^14^ Counts were assigned to individual cells based on cell segmentation borders. Cells with fewer than 20 counts or with area more than 5 times the average cell area were removed. Data were normalized to total counts per cell and square root transformed. Uniform Manifold Approximation and Projection (UMAP) dimensionality reduction was then performed using all genes. Unsupervised Leiden clustering was run with 50 principal components and a resolution of 0.4. The following analysis was performed using Seurat^8,9^ unless otherwise noted. Differential expression using FindAllMarkers with min.pct = 0.25 was used to identify cluster markers genes. Clusters were annotated manually based on known marker genes. For niche analysis, the cellular composition within a 50-μm radius of each cell was determined and clustered into 7 niches using MClust.^15^ Differential expression between fibroblasts based on niche or distance to neutrophils was performed using FindMarkers with min.pct = 0.25.

### Cell culture

Healthy human dermal fibroblasts were isolated from 2 × 4-mm skin punches by mechanical digestion with scissors and enzymatic digestion with 0.2% collagenase for 30 minutes at 37°C. Cells were grown in a humidified incubator at 5% CO_2_ and 37°C under sterile conditions and used at passage 3. Cells were grown in RPMI medium supplemented with L-glutamine, 10% FBS, and antibiotic-antimycotic (Thermo Fisher Scientific [Waltham, Mass]; catalog no. 15240062). Cells at 80% confluency were stimulated with recombinant cytokines including rhIFNa2 (R&D Systems [Minneapolis, Minn]; catalog no. 11100-1; dose 5 ng/mL), rhTNF-α (R&D Systems; catalog no. 210-TA-005; dose 10 ng/mL), or rhIFN-γ (R&D Systems; catalog no. 285-IF-100; dose 5 ng/mL). After 6 hours of stimulation, cells were lysed with RNA lysis buffer (Qiagen [Hilden, Germany]; catalog no. 74104).

### RNA isolation protocol

For bulk RNA-Seq and quantitative PCR (qPCR) of FFPE tissue, RNA was isolated from FFPE tissue using the RNeasy DSP FFPE Kit (Qiagen; catalog no. 73604). For bulk RNA-Seq and qPCR of cultured fibroblasts, RNA was isolated from cell lysates using the RNeasy kit (Qiagen; catalog no. 74104).

### ELISA

Cultured fibroblasts were stimulated with IFNa2 (5 ng/mL, Peprotech [Cranberry, NJ]; catalog no. 300-02AA) or vehicle control. After 72 hours, cell supernatant was used to measure IL-8/CXCL8 secretion by ELISA according to the manufacturer’s protocol (R&D Systems; catalog no. DY208).

### Bulk RNA-Seq

Libraries were generated using a QuantSeq 3′ mRNA-Seq Library Prep Kit (Lexogen) and sequenced using a NovaSeq (Illumina). After adaptor trimming, reads were mapped to the hg38 reference genome using STAR, count matrices were generated using HTSeq, and differential expression analysis was performed using DESeq2. Volcano plots and heatmaps were generated using EnhancedVolcano and pheatmap. Pathway analysis was performed with Metascape.^12^ Machine learning cell type deconvolution was performed using CIBERSTORTx^16^ with the following settings: S-mode batch correction, 100 permutations, quantile normalization disabled, and relative mode. snRNA-Seq data were downsampled to 5000 cells (250 cells per cluster) and used as the reference data set.

### Quantitative RT-PCR

RNA was converted to cDNA using a High-Capacity cDNA Reverse Transcription Kit (Thermo Fisher Scientific; catalog no. 4368814). qPCR was performed using a QuantStudio Real-Time PCR system (Thermo Fisher Scientific) with TaqMan Universal PCR Master Mix (Thermo Fisher Scientific; catalog no. 4304437). Housekeeping gene *RPLP0* was used to normalize expression. The TaqMan primers included *RPLP0* (Thermo Fisher Scientific; catalog no. Hs004200895_gh), *CXCL1* (Thermo Fisher Scientific; catalog no. Hs00236937_m1), *CXCL2* (Thermo Fisher Scientific; catalog no. Hs00234140_m1), *CXCL3* (Thermo Fisher Scientific; catalog no. Hs00171061_m1), *CXCL5* (Thermo Fisher Scientific; catalog no. Hs00982282_m1), *CXCL6* (Thermo Fisher Scientific; catalog no. Hs00237017_m1), and *CXCL8* (Thermo Fisher Scientific; catalog no. Hs00174103_m1).

### Immunofluorescence

Antigen retrieval of FFPE sections was performed using Target Retrieval Solution (Dako [Santa Clara, Calif]; catalog no. S2369) as per manufacturer recommendations. Sections were blocked with serum from secondary antibody host and stained with primary antibodies overnight at 4°C and secondary antibodies for 1 hour at room temperature; nuclei were counterstained with 4′,6-diamino-2-phenylindole. Epifluorescence images were taken using an EVOS5000. Brightness and contrast were adjusted slightly using ImageJ or Nikon elements software and applied equally across samples. The primary antibodies used were CXCL1 (Thermo Fisher Scientific; catalog no. PA586508, 1:100) and CXCL12 (Thermo Fisher Scientific; catalog no. 14-7992-81, 1:1000); the secondary antibody was Cy3 Donkey anti-Rabbit IgG (BioLegend [San Diego, Calif]; catalog no. 406402, 1:500). Quantification was performed using the plot profile function in ImageJ.

### Statistical analysis

snRNA-Seq and spatial transcriptomics differential expression analysis were performed using Seurat. Bulk RNA-Seq differential expression analysis was performed using DESeq2. Transcriptomics *P* values were adjusted for multiple hypothesis testing, with adjusted *P* values less than .05 considered statistically significant. qPCR statistical significance was calculated using GraphPad Prism (GraphPad Software, San Diego, Calif) (with *P < .05; **P < .01; ***P < .001; and ****P < .0001).

### Study approval

Human skin biopsy samples for spatial transcriptomics, hematoxylin and eosin staining, and immunofluorescence were collected from the University of California San Diego Dermatology Clinic. Sample acquisitions were approved and regulated by the University of California San Diego institutional review board (approval no. 140144). Acquisition of human skin samples for snRNA-Seq, bulk RNA-Seq, and *in vitro* fibroblast studies was approved by University of Michigan institutional review board. Written informed consent was obtained from all subjects.

### Data availability

The genomic data presented here are available in the National Center for Biotechnology Information Gene Expression Omnibus database under reference series GSE299256 (available at https://www.ncbi.nlm.nih.gov/geo/query/acc.cgi?acc5GSE299256).

## RESULTS

### Activation by interferon is a prominent feature of several SS skin cell subsets, including fibroblasts

**FIG 1.**
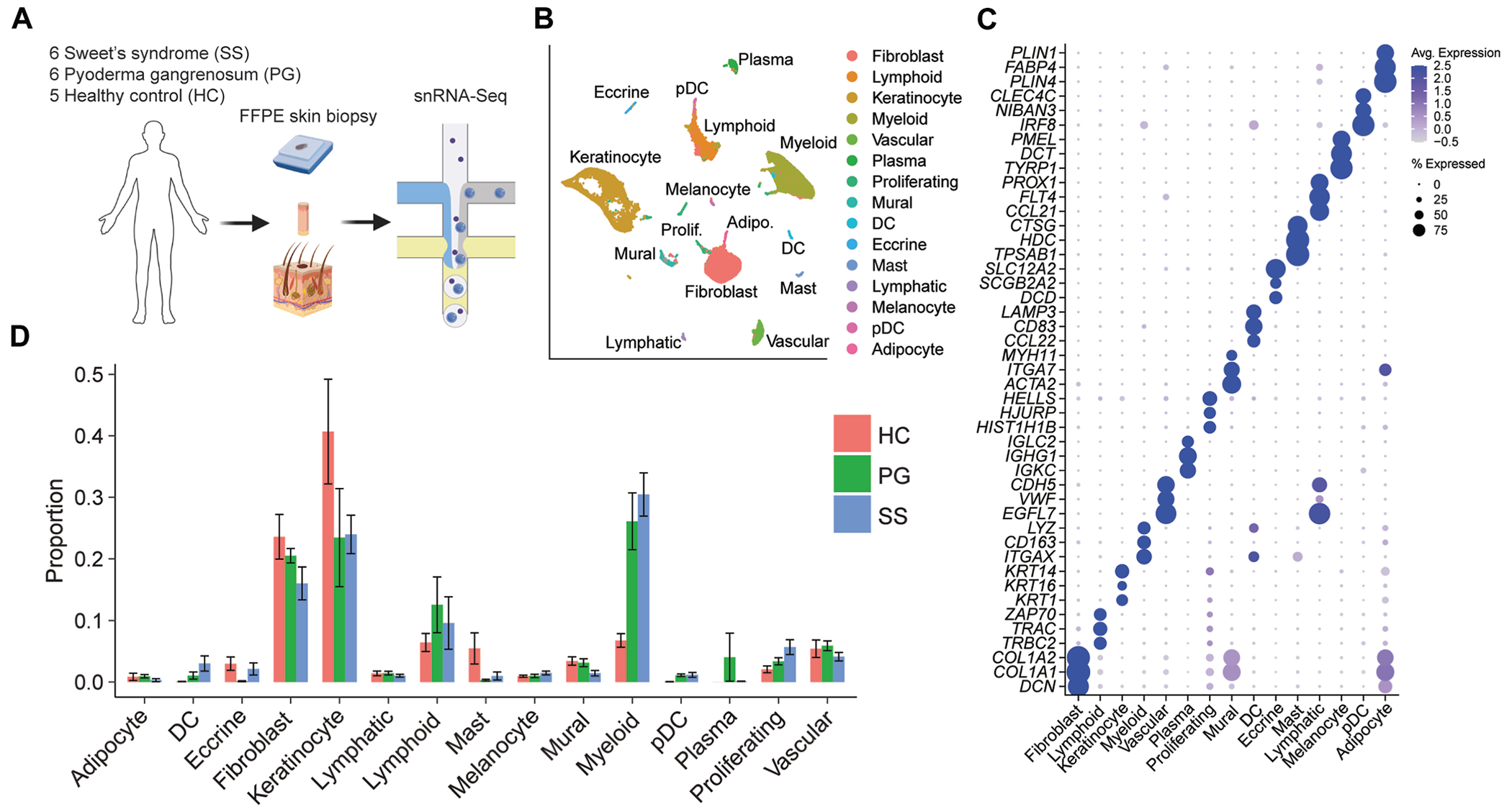
Single-nucleus RNA sequencing of skin from patients with neutrophilic dermatoses and HCs. **A,** Experimental schematic. **B,** Dimensionality reduction and unsupervised clustering, colored by cell type. **C,** Expression of the top 3 marker genes per cell type. **D,** Proportion of cell types for each condition. Error bars represent SEMs. *Adipo.*, Adipocyte; *Prolif.*, proliferating.

To understand the cellular landscape of SS, we performed single-nucleus transcriptomics using the 10X Genomics Flex platform on archived clinical FFPE lesional skin biopsy samples from patients with SS (n = 6). Analysis was also performed on skin from patients with another neutrophilic dermatosis, namely, early-stage PG (n = 6), as well as on skin from HCs (n = 5), to identify potentially unique gene signatures within SS (Fig 1, A). In total, we recovered 70,534 nuclei, with an average of 1,088 genes and 1,706 unique transcripts detected per nucleus (see Fig E1, A in the Online Repository at www.jacionline.org). Quality control pseudobulk principal component analysis confirmed that the neutrophilic dermatoses samples were transcriptionally distinct from HC samples (see Fig 1, B in the Online Repository at www.jacionline.org).

Recovered nuclei were annotated using known marker genes following dimensionality reduction and unsupervised clustering (Fig 1, B), revealing 15 major cell types (Fig 1, C): fibroblasts (*COL1A1, COL1A2*, and *DCN*), lymphoid cells (*TRAC, TRBC2*, and *ZAP70*), keratinocytes (*KRT14, KRT16*, and *KRT1*), myeloid cells (*ITGAX, CD163*, and *LYZ*), vascular endothelial cells (*EGFL7, VWF*, and *CDH5*), plasma cells (*IGKC, IGHG1*, and *IGLC2*), proliferating cells (*HIST1H1B, HJURP*, and *HELLS*), mural cells (comprising pericytes and smooth muscle cells; *ITGA7, MYH11*, and *ACTA2*), dendritic cells (DCs) (*CCL22, CD83*, and *LAMP3*), eccrine cells (*DCD, SCGB2A2*, and *SLC12A2*), mast cells (*TPSAB1, HDC*, and *CTSG*), lymphatic endothelial cells (*CCL21, FLT4*, and *PROX1*), melanocytes (*DCT, TYRP1*, and *PMEL*), plasmacytoid DCs (pDCs) (*NIBAN3, CLEC4C*, and *IRF8*), and adipocytes (*PLIN1, FABP4*, and *PLIN4*). Proliferating cells included lymphoid cells, fibroblasts, keratinocytes, and myeloid cells (see Fig E1, C and D). Myeloid cells (quiescent and proliferating), proliferating fibroblasts, lymphoid cells, DCs, and pDCs were enriched in neutrophilic dermatoses lesions versus HC skin (Fig 1, D and see Fig E1, E).

**FIG 2.**
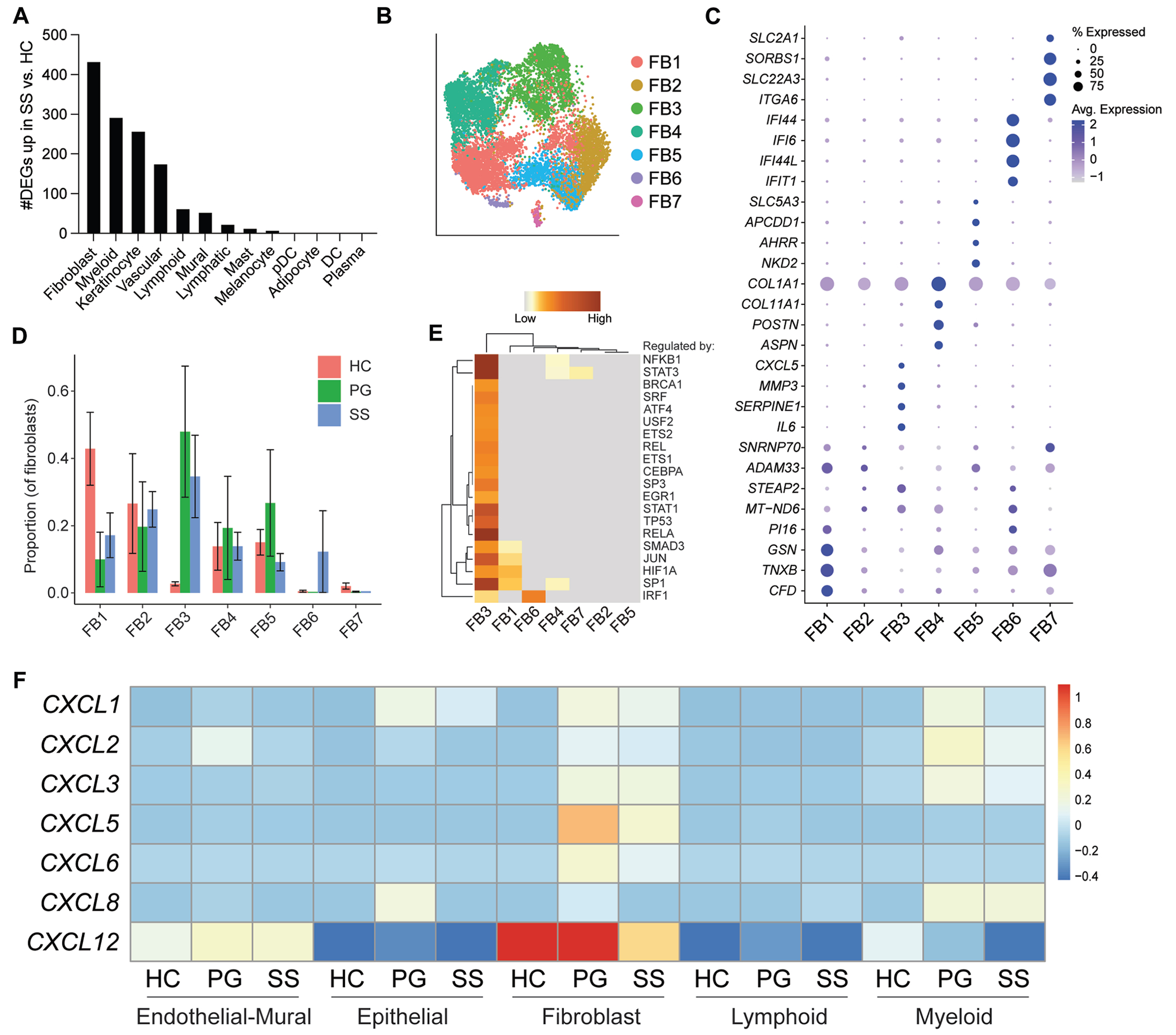
Interferon (IFN)-activated fibroblasts (FBs) are enriched in SS skin. **A,** Number of significantly upregulated genes (adjusted *P* value [*P*_adj_] < .05) in patients with SS versus patients with HC for each cell type. Differential expression analysis was performed using DESeq2. Pseudobulked data were used to control for cell number. **B,** Dimensionality reduction and unsupervised clustering of FBs, colored by subset. **C,** Expression of the top 4 marker genes per FB subset. **D,** Proportion of FB subsets for each condition. **E,** Pathway analysis inferring upstream transcription factors regulating gene expression for each cluster. **F,** Scaled expression of neutrophil chemokine CXCR2 ligands (*CXCL1, CXCL2, CXCL3, CXCL5, CXCL6*, and *CXCL8*) and CXCR4 ligand (*CXCL12*) across cell types for each condition. Keratinocytes, eccrine cells, and melanocytes were combined into the category epithelial. DCs, pDCs, mast cells, and myeloid cells were combined into the category myeloid. Plasma cells and lymphoid cells were combined into the category lymphoid. Vascular endothelial cells, lymphatic endothelial cells, and mural cells were combined into the category endothelial-mural. Proliferating cells and adipocytes were excluded. Error bars represent SEMs.

To understand which cell types may drive skin inflammation in SS, we determined the extent of transcriptional response of each cell type by performing differential expression analysis between HC skin and SS skin (Fig 2, A). Pseudobulk data were used to control for cell number. This analysis revealed that fibroblasts were the most transcriptionally activated cell type in SS, followed by myeloid cells and then keratinocytes.

Dimensionality reduction and unsupervised clustering of fibroblasts identified 7 transcriptionally distinct fibroblast subsets (referred to as FB1-FB7) (Fig 2, B and C). FB3 was enriched in both neutrophilic dermatoses versus HC skin, and it was marked by expression of neutrophil chemokines and proinflammatory cytokines (*CXCL5* and *IL6*) (Fig 2, D). FB4 and FB5 were expanded in PG skin and highly expressed markers for myofibroblasts (*COL1A1* and *COL1A11*) and papillary fibroblasts (*APCDD1*), respectively. FB6, which was marked by interferon-induced genes (*IFI44, IFI6, IFI44L*, and *IFIT1*), was found almost exclusively in SS skin. FB1 highly expressed the universal fibroblast gene *PI16*^17^ and was decreased in frequency in both disease conditions, hinting that this subset may serve as a progenitor for neutrophilic dermatosis–associated fibroblast subsets. Pathway analysis suggested that FB3 and FB6 are regulated by interferon response factor 1 (IRF1); therefore, these subsets may both be induced by interferon (Fig 2, E).

Compared with other cell lineages, fibroblasts in SS skin and PG skin were a dominant source of neutrophil chemokines that bind CXCR2 (*CXCL1, CXCL2, CXCL3, CXCL5, CXCL6*, and *CXCL8*) and CXCR4 (*CXCL12*) (Fig 2, F). Taken together, these results demonstrate that SS is associated with a prominent interferon signature and suggest that fibroblast recognition of interferon may drive neutrophil recruitment in SS.

Next, lymphoid, myeloid, and keratinocyte subsets were interrogated to understand whether other cell types respond to interferon in SS (see Fig E2, A–C in the Online Repository at www.jacionline.org). A total of 9 transcriptionally distinct lymphocyte subpopulations (referred to as LYM1-LYM9) were identified by dimensionality reduction and unsupervised clustering of the initial lymphoid and plasma cell clusters (see Fig E2, A). The levels of regulatory T cells LYM6 (*FOXP3*) and interferon-activated lymphocytes LYM8 (*MX1, OAS3*, and *IFI44L*) were elevated in patients with SS compared with HCs and patients with PG (see Fig E2, D). The latter was found almost exclusively in patients with SS. B cells and plasma cells LYM2 (*IGLC3, IGHG2*, and *IGKC*) and LYM9 (*IGHM* and *IGLC1*), tissue-resident memory T cells LYM5 (*CD69*), and stem-like CD4 T cells LYM1 (*IL7R* and *TCF7*) were enriched in patients with PG versus patients with SS and HCs. The levels of CD8^+^ T cells LYM4 (*CD8A*) and natural killer cells LYM7 (*KLRD1*) were decreased in frequency in patients with PG versus HCs and patients with SS.

We next investigated keratinocyte subsets. Using dimensionality reduction and unsupervised clustering, we found 10 transcriptionally discrete clusters (referred to as KC1-KC10) (see Fig E2, B). The frequencies of basal keratinocytes (KC1 [*KRT15*]) and suprabasal keratinocytes (KC3 [*KRT1* and *KRT10*]) were decreased in both neutrophilic dermatoses, whereas the frequencies of stratum granulosum keratinocytes (KC2 [*KLK13*)]) and interferon-activated keratinocytes (KC8 [*IRF1, WARS*, and *CXCL10*]) were increased in patients with SS and PG (see Fig E2, F). No major differences were observed between the keratinocytes in patients with SS and those with PG.

Dimensionality reduction and unsupervised clustering of the initial myeloid, pDC, DC, and mast cell clusters resolved 10 transcriptionally distinct myeloid cell clusters (referred to as MY1-MY10) (see Fig E2, C). MY4 mast cells (*GATA2, HDC*, and *TPSAB1*) were enriched in HC skin, whereas the MY6 DCs (*CCR7* and *CCL22*), MY7 pDCs (*GZMB*), and TREM2 macrophages MY5 (*SPP1*) were enriched in both neutrophilic dermatoses (see Fig E2, F). Interferon-activated myeloid cells MY1 (*CXCL10, GBP5*, and *CD300E*) were abundant in patients with SS but not in those with PG or in HCs. We were unable to confidently annotate a bona fide neutrophil cluster. Pathway analysis indicated that LYM8, KC8, and MY1 may be regulated by IRF1 and/or STAT1, confirming the identity of these subsets as interferon-activated (see Fig E2, G–I). Thus, interferon-activated subsets were expanded in all major cell lineages in SS skin.

We next determined the interferon signature in individual patients with SS. Each SS patient sample possessed a unique composition of interferon-activated cell types (see Fig E3, A–E in the Online Repository at www.jacionline.org). Interferon-activated myeloid cells were observed in all patients with SS to a varying degree, whereas interferon-activated fibroblasts, lymphocytes, and keratinocytes were identified in a subset of patients. These results demonstrate that the prominent interferon signature is a conserved feature of SS, although the cell type on which interferon acts is patient dependent.

**FIG 3.**
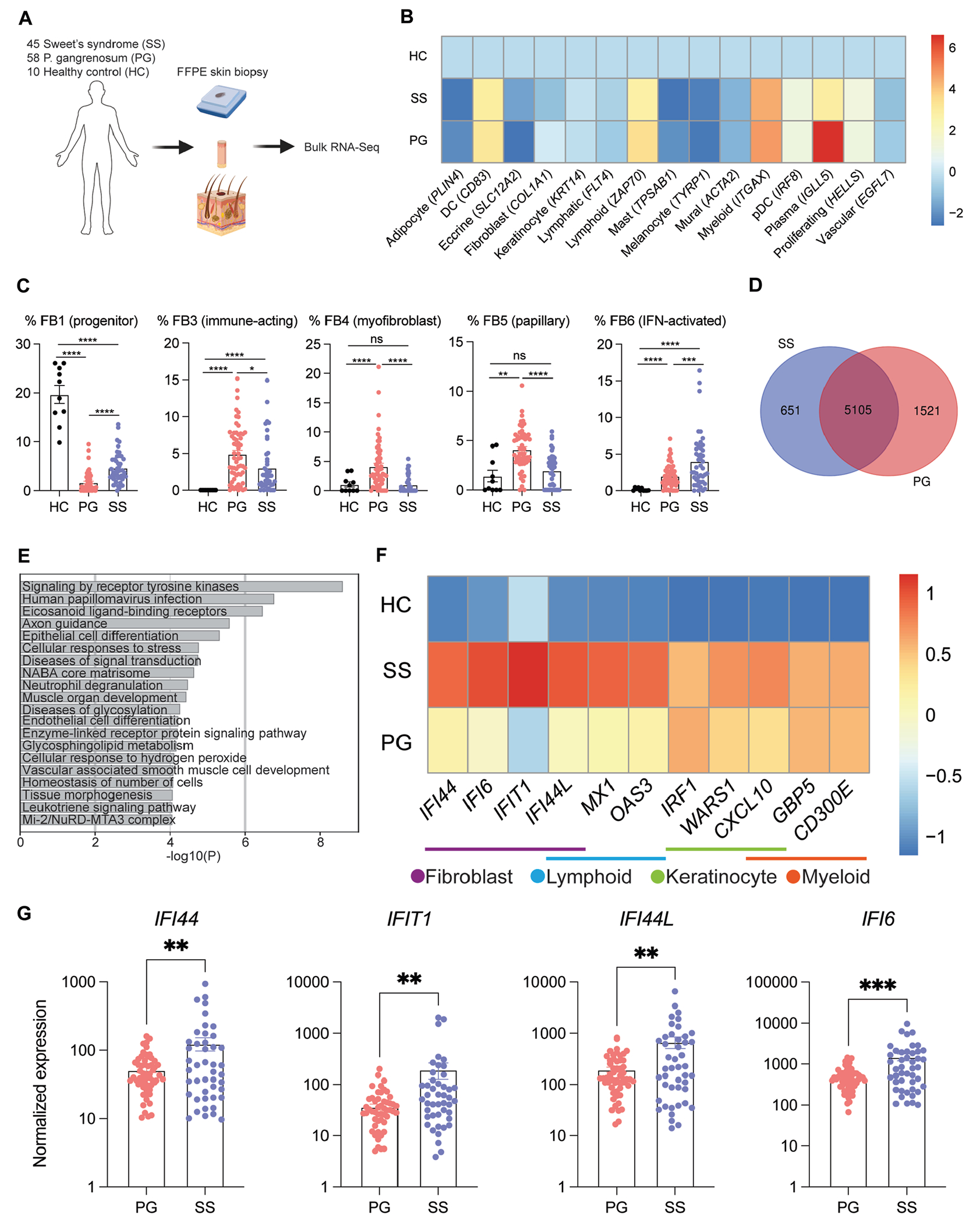
Bulk RNA-Seq of neutrophilic dermatoses and HC skin validates prominent interferon signature in SS. **A,** Experimental schematic. **B,** Cell type deconvolution of bulk RNA-Seq using unique marker genes from snRNA-Seq. Values represent log_2_ fold changes in samples from HCs versus samples from patients with SS and HCs versus samples from patients with PG. **C,** Frequency of fibroblast (FB) subsets by machine learning–based cell type deconvolution with CIBERSORTx. Each dot represents 1 patient. **D,** Number of differentially expressed genes (up and down [adjusted *P* value (*P*_adj_) < .05]) in patients with SS and patients with PG compared with HCs. **E,** Pathway analysis with the 651 differentially expressed genes unique to SS. **F,** Bulk RNA-Seq expression of interferon (IFN)-induced genes marking each cellular compartment in snRNA-Seq data, scaled by column. Values represent log_2_ fold changes from HCs versus patients with SS and HCs versus patients with PG. **G,** Bulk RNA-Seq expression of IFN-induced genes marking IFN-activated FB6. Each dot represents 1 patient. *P < .05; **P < .01; ***P < .001; and ***P < .0001 using unpaired *t* tests. Error bars represent SEMs.

To validate our snRNA-Seq findings, we next performed bulk RNA-Seq on a large independent cohort of samples of SS (n = 45), PG (n = 58), and HC skin (n = 10) (Fig 3, A). Cell type deconvolution using unique cell type marker genes confirmed that the numbers of DCs, lymphoid cells, myeloid cells, pDCs, plasma cells, and proliferating cells are increased in neutrophilic dermatoses versus healthy skin (Fig 3, B). A more granular machine learning–based deconvolution analysis was performed with CIBERSORTx^16^ to quantify the frequency of fibroblast subsets. This analysis indicated that interferon-activated FB6 was significantly elevated in SS compared with PG skin and HC skin, with the frequency of FB6 in SS skin elevated above the average FB6 frequency in PG skin in 26 of 45 patients (Fig 3, C). Additionally, progenitor FB1 was enriched in HCs, immune-acting FB3 was increased in skin from patients with both neutrophilic dermatoses, and myofibroblast FB4 and papillary FB5 were elevated in PG. These results confirm snRNA-Seq findings and demonstrate that interferon-activated fibroblasts are found in a large subset of patients with SS.

Comparative differential expression analysis identified 651 and 1521 genes uniquely regulated in SS and PG, respectively, and 5105 similarly regulated genes (Fig 3, D). The proinflammatory cytokines *IL1A*, *IL1B*, *TNF*, and *IL-6* and neutrophil chemokines *CXCL1, CXCL2, CXCL3, CXCL5, CXCL6*, and *CXCL8* were significantly upregulated in patients with SS and patients with PG compared with HCs (see Fig E4, A and B in the Online Repository at www.jacionline.org). *IL17A* was not significantly upregulated in patients with PG or SS. The genes upregulated in both diseases were consistent with an innate immune response and neutrophil degranulation (see Fig E4, C). Pathway analysis suggested that PG-specific genes, including the myofibroblast marker *COL11A1*, were consistent with developmental processes and extracellular matrix production, validating the snRNA-Seq findings (see Fig E4, D). SS-specific genes were consistent with human papilloma virus infection—a type I interferon response pathway (Fig 3, E). Expression of the 11 interferon-stimulated genes defining interferon-activated subsets of fibroblasts, myeloid cells, lymphoid cells, and keratinocytes was significantly increased in patients with SS versus HCs (Fig 3, F). Expression of 10 of these genes—particularly those marking interferon-activated fibroblasts and lymphocytes—was increased in SS versus PG. Expression of genes marking interferon-activated fibroblasts, *IFI44, IFI6, IFI44L*, and *IFIT1*, was significantly increased in SS versus PG (Fig 3, G). Taken together, the findings of this unbiased analysis of a large independent patient cohort validate the prominent interferon signature and enrichment of interferon-activated fibroblasts identified in SS by snRNA-Seq.

### Type I interferon activates primary human dermal fibroblasts to highly express neutrophil chemokines

**FIG 4.**
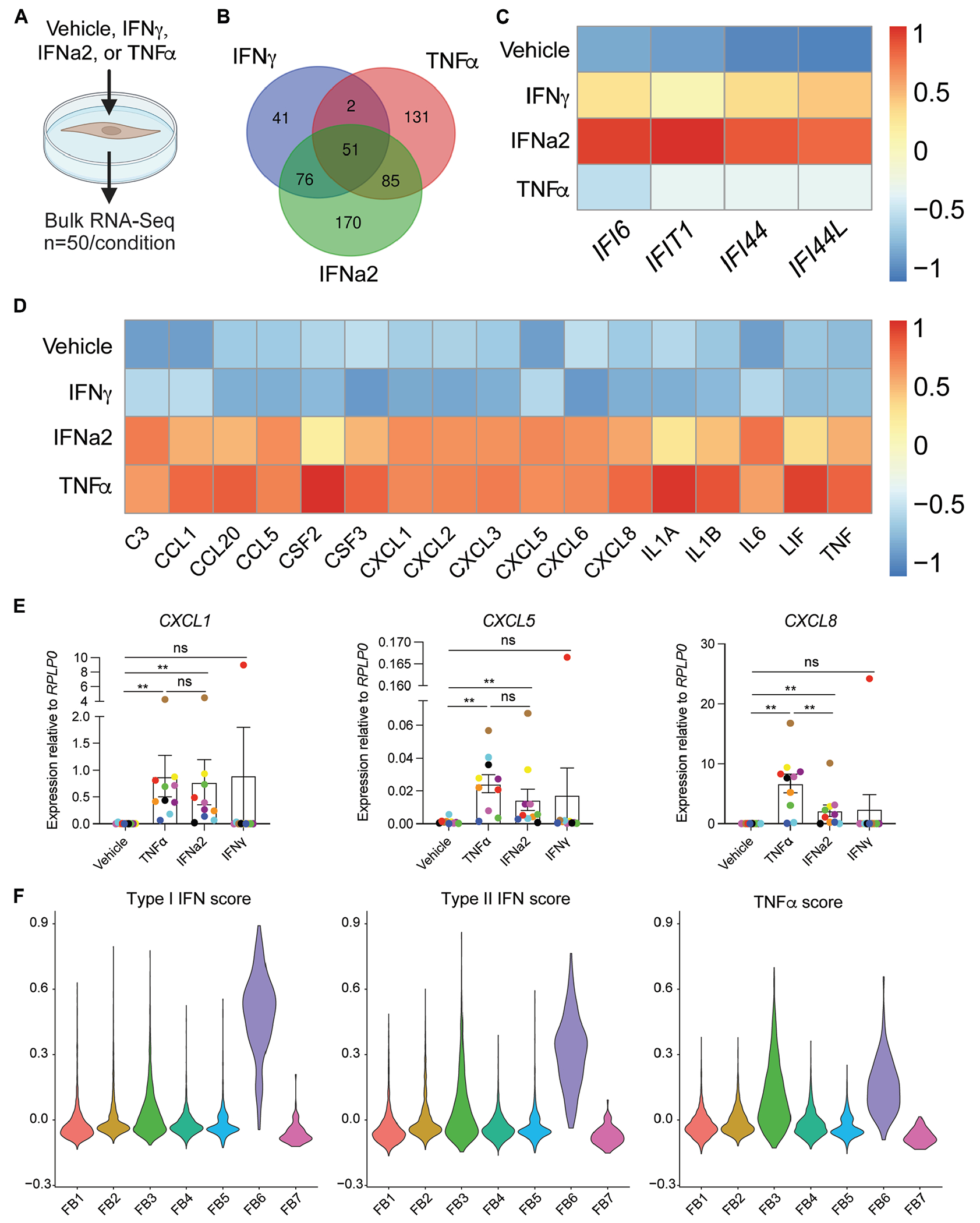
Cultured primary human dermal fibroblasts (FBs) are activated by type 1 interferon (IFN) to express neutrophil chemokines. **A,** Schematic of *in vitro* assay to test significance of IFN signature in primary human dermal FBs. **B,** Venn diagram comparing the number of genes highly upregulated (adjusted *P* value [*P*_adj_] < .05; log_2_ fold change > 3) per stimulus. **C,** Bulk RNA-Seq–normalized gene expression of IFN-induced genes marking FB6 that were highly upregulated by IFN-γ and IFNa2 but not by TNFα. **D,** Bulk RNA-Seq–normalized gene expression of select neutrophil chemokines, myeloid growth factors, and proinflammatory cytokines that were highly upregulated by TNF-α and IFNa2 but not by IFN-γ. **E,** qPCR expression of neutrophil chemokines in stimulated dermal FBs. Each color represents a single donor. **F,** IFN-γ, IFNa2, and TNF-α signature scores in snRNA-Seq FB subsets using all significantly upregulated genes (*P*_adj_ < .05) by each cytokine *in vitro*. *P < .05; **P < .01; ***P < .001; and ****P < .0001 using the Wilcoxon matched-pairs signed rank test. Error bars represent SEMs. *ns*, Not significant.

To test the significance of the interferon signature in fibroblasts for neutrophil recruitment, we cultured primary human dermal fibroblasts from 50 different donors with type I interferon (IFNa2) and type 2 IFN-γ and examined gene expression by bulk RNA-Seq (Fig 4, A). The effect of these signals was compared with that of TNF-α, which has been shown to induce fibroblast expression of neutrophil chemokines.^18,19^ Comparative differential expression analysis identified 76 genes highly upregulated by IFN-γ and IFNa2 but not by TNF-α (Fig 4, B). Among these genes were the interferon-stimulated genes that marked interferon-activated FB6 (Fig 4, C). Highly upregulated by all 3 stimuli were chemokines for monocytes/macrophages (*CCL2, CCL7, CCL8*, and *CX3CL1*) and lymphocytes (*CXCL9, CXCL10*, and *CXCL11*) (see Fig E5, A in the Online Repository at www.jacionline.org). Few inflammatory genes were highly upregulated by IFN-γ alone (*CCL13*) or by TNF-α alone (*IL34, TSLP*). Remarkably, the 85 genes that were highly upregulated by TNF-α and IFNa2 but not by IFN-γ included neutrophil chemoattractants (*CXCL1*, *CXCL5*, *CXCL8*, and *C3*), myeloid cell growth factors (*CSF2* and *CSF3*), and proinflammatory cytokines associated with SS (*IL1A, IL1B, IL6*, and *TNF*) (Fig 4, D). Neutrophil chemokine upregulation by type I interferon was validated at the transcript level by qPCR (Fig 4, E and see Fig E5, B) and the protein level by ELISA on cell supernatant (see Fig E5, C).

To infer whether type I or type II interferon drove the interferon signature in FB6, *in vitro* activation gene modules were generated and projected onto snRNA-Seq data. This analysis indicated that the transcriptomic signature of interferon-activated FB6 was most consistent with activation by type I interferon (Fig 4, F).

We next sought to understand the sources of type I interferon in SS. snRNA-Seq revealed a 15-fold increase in pDCs in neutrophilic dermatoses versus normal skin (see Fig E6, A in the Online Repository at www.jacionline.org). pDCs are well known to produce large amounts of type I interferon.^20^ Bulk RNA-Seq deconvolution confirmed that the numbers of pDCs were significantly increased in patients with SS and patients with PG versus HCs (see Fig E6, B). Notably, the frequencies of pDCs and interferon-activated FB6 were positively correlated in SS, providing further evidence that pDCs may be an important source of type I interferon (see Fig E6, C). No statistical difference was observed between the frequency of pDCs in patients with SS and those with PG, suggesting that pDCs may be more activated to produce type I interferon in SS than in PG (see Fig E6, A and B). To understand where pDCs reside in the skin of patients with neutrophilic dermatoses, we performed immunostaining for CD123, a highly specific marker of pDCs.^21^ Increased CD123 staining was observed in SS and PG skin, localized to the area surrounding the dermal neutrophilic infiltrate (see Fig E6, D). Taken together, these findings support a model in which pDCs contribute to increased type I interferon, which then activates subsets of fibroblasts in SS for neutrophil recruitment.

### Spatially distinct fibroblast subsets promote neutrophil recruitment in SS

**FIG 5.**
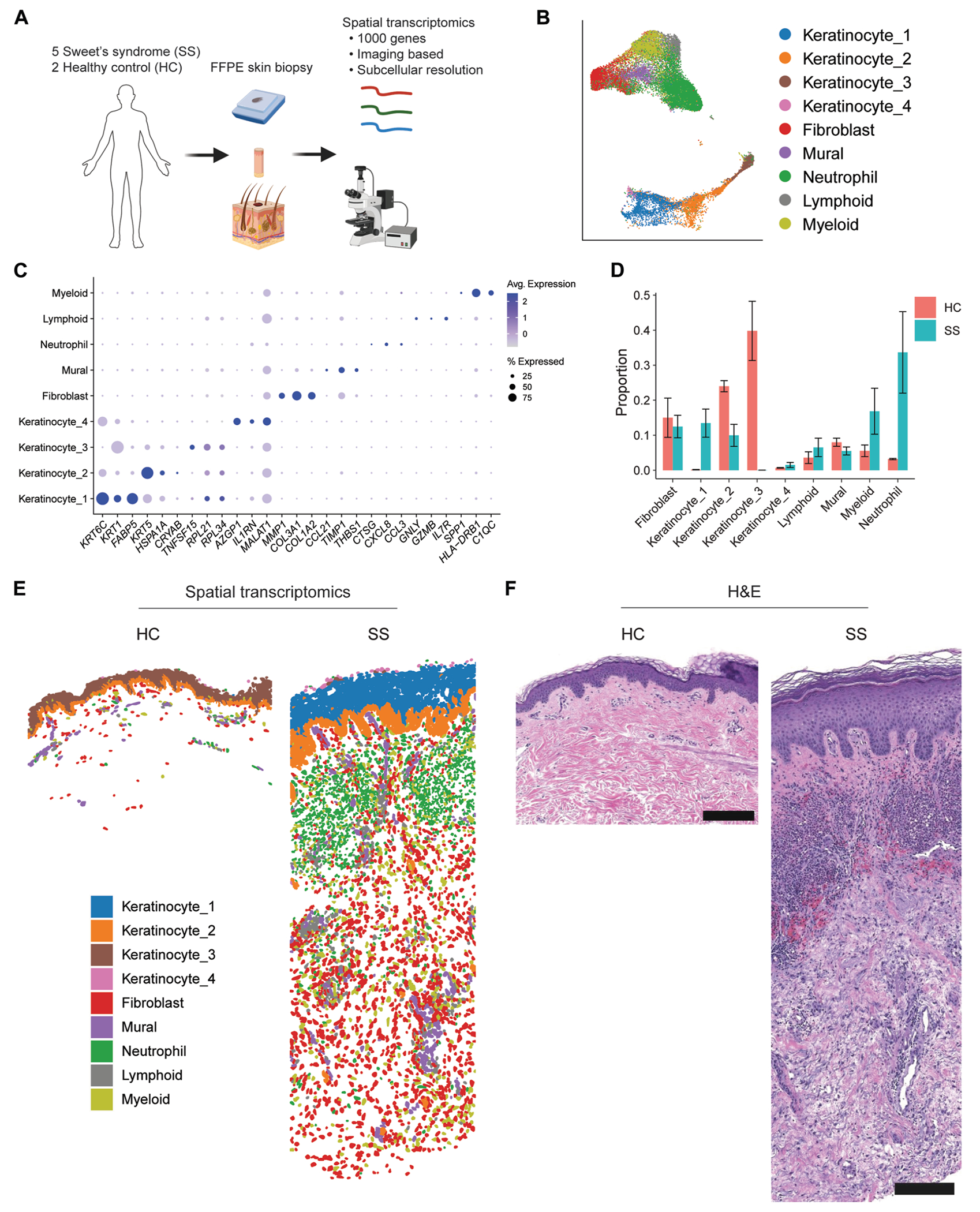
Subcellular resolution spatial transcriptomics of SS and HC skin. **A,** Experimental schematic using the Nanostring CosMx subcellular resolution spatial transcriptomics platform. **B,** Dimensionality reduction and unsupervised clustering, colored by cluster. **C**, Expression of the top 3 marker genes per cluster. **D,** Proportion of clusters for each condition. **E,** Clusters projected onto representative tissue sections from HCs (HC 2) and patients with SS (patient with SS 4). **F,** Hematoxylin and eosin (H&E) staining of adjacent tissue sections from HCs and patients with SS. Scale bar = 200 μm. Error bars represent SEMs.

We next sought to understand the cellular and molecular landscape of neutrophilic dermatoses biogeographically. To this end, we performed subcellular resolution spatial transcriptomics on archival FFPE skin biopsy samples from patients with SS (n = 5) and HCs (n = 2) using Nanostring’s 1000-gene imaging-based CosMx platform (Fig 5, A). At the single-cell level, our data set included 26,792 cells, with an average of 47 genes and 94 transcripts detected per cell. Dimensionality reduction and unsupervised clustering revealed 9 transcriptionally distinct clusters (Fig 5, B and C): neutrophils (*CTSG, CXCL8*, and *CCL3*), keratinocyte cluster 1 (*KRT6C, KRT1*, and *FABP5*), keratinocyte cluster 2 (*KRT5, HSPA1A*, and *CRYAB*), keratinocyte cluster 3 (*TNFSF15, RPL21*, and *RPL34*), keratinocyte cluster 4 (*AZGP1, IL1RN*, and *MALAT1*), fibroblasts (*MMP1, COL3A1*, and *COL1A2*), myeloid cells (*SPP1, HLA-DRB1*, and *C1QC*), lymphoid cells (*GNLY, GZMB*, and *IL7R*), and mural cells (*CCL21, TIMP1*, and *THBS1*). The frequencies of myeloid cells and lymphoid cells were increased in patients with SS versus HCs (Fig 5, D and see Fig E7, A in the Online Repository at www.jacionline.org), thus confirming our snRNA-Seq and bulk RNA-Seq findings.

The cell clusters were then projected onto tissue sections to elucidate spatial localization (Fig 5, E and see Fig E7, B–L). Strikingly, all of the unsupervised clusters mapped to unique histologic regions. The patients with SS and the HCs shared basal epidermal keratinocyte cluster 2 but had distinct spinous and supraspinous epidermal clusters. Unlike single-cell and single-nucleus transcriptomics, which often fail to recover neutrophils, spatial transcriptomics identified a cluster of neutrophils that was validated by hematoxylin and eosin staining (Fig 5, F). The neutrophils in patients with SS were observed to be densely packed in the mid-to-upper dermis.

**FIG 6.**
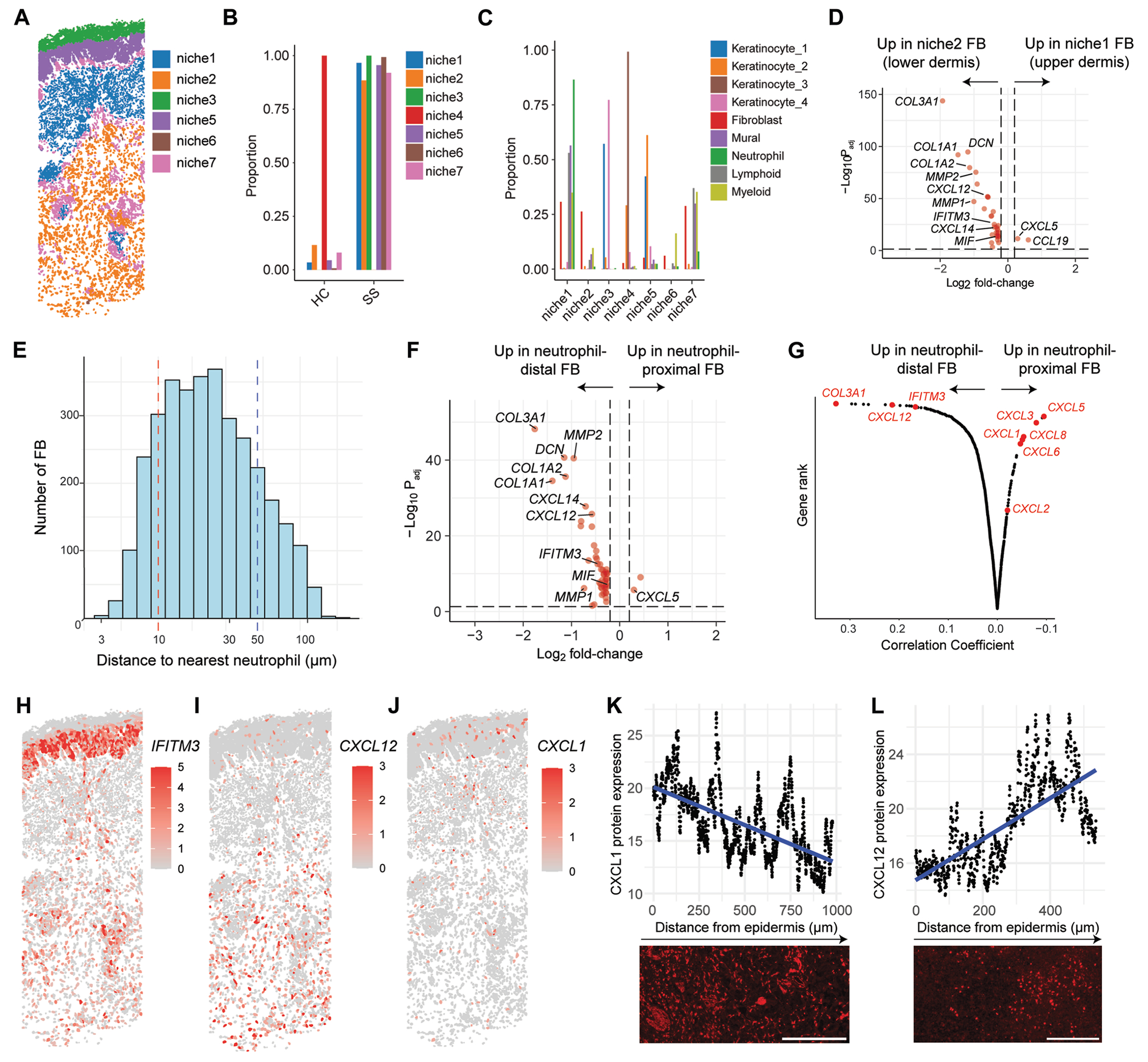
Niche analysis reveals positionally distinct immune-acting fibroblasts (FBs) in SS skin. **A,** Niche analysis projected onto SS tissue. **B,** Proportion of each niche per condition. **C,** Proportion of each cell type per niche (includes all conditions). **D,** Differential expression analysis between FBs in upper dermal niche 1 and FBs in lower dermal niche 2. Adjusted *P* value (*P*_adj_) threshold = .05. Log_2_ fold change (FC) threshold = 0.2. **E,** Distribution of FB distance to nearest neutrophil. **F,** Differential expression between neutrophil proximal FBs (<30 μm) and neutrophil distal FBs (>300 μm). *P*_adj_ threshold = .05. Log_2_ fold change (FC) threshold = 0.2. **G,** Rank order of genes based on correlation with FB distance to nearest neutrophil. SS expression of interferon-induced gene *IFITM3* (**H**) and neutrophil chemokines *CXCL12* (**I**) and *CXCL1* (**J**). **K,** Protein immunostaining of CXCL1 in SS (*bottom*) and quantification of expression as a function of distance from epidermis (*top*). Scale bar = 300 μm. The experiment was conducted twice. **L,** Protein immunostaining of CXCL12 in SS (*bottom*) and quantification of expression as a function of distance from epidermis (*top*) Scale bar = 150 μm. The experiment was conducted twice.

We next used MClust to perform unsupervised niche analysis by clustering cells into 7 regions based on the cellular composition within a 50-μm radius of each cell.^15^ This analysis resolved an SS-associated upper dermal niche (niche 1) and an SS-associated lower dermal niche (niche 2) (Fig 6, A and B and see Fig E8, A). Niche 1 included fibroblasts and neutrophils, whereas niche 2 included fibroblasts but no neutrophils (Fig 6, C). Differential expression analysis between the fibroblasts in niche 1 and those in niche 2 indicated that fibroblasts in the upper dermal, neutrophil-proximal niche were marked by *CCL19* the interferon-inducible neutrophil chemokine gene *CXCL5*, whereas fibroblasts in the lower dermal, neutrophil-distal niche were marked by the interferon-inducible gene *IFITM3*, the neutrophil chemokine genes *CXCL12* and *MIF*, and extracellular matrix genes (*COL3A1, COL1A1, COL1A2*) (Fig 6, D). Similar results were obtained by performing differential expression analysis between neutrophil-proximal (<10 μm) and neutrophil-distal (>50 μm) fibroblasts and by correlating fibroblast gene expression with neutrophil distance (Fig 6, E–G). Projection of *IFITM3*, *CXCL1*, and *CXCL12* expression onto tissue sections indicated that these genes were not expressed in HC skin but were highly expressed in SS lesions (Fig 6, H–J and see Fig E8, B–D). *IFITM3* demonstrated striking regional expression in the epidermis, with high expression in basal and lower suprabasal keratinocytes but not in upper suprabasal keratinocytes (Fig 6, H and see Fig E8, E). Protein immunostaining of SS skin confirmed that CXCL1 and CCL19 expression negatively correlates with distance from epidermis and that CXCL12 expression positively correlates with distance from epidermis (Fig 6, K and L and see Fig E8, F). Thus, unbiased subcellular resolution spatial transcriptomics identified positionally distinct dermal IAF subsets in SS lesions.

## DISCUSSION

In this study, we aimed to develop a comprehensive cellular and molecular atlas of the human neutrophilic dermatosis SS—a rare disease not previously profiled at scale by using unbiased transcriptomics. Comparative analysis of single-nucleus transcriptomics of archival clinical skin biopsy samples from patients with SS, patients with early PG, and HCs revealed new cellular contributors to neutrophilic dermatoses, including pDCs. This analysis also led to the identification of an interferon-activated fibroblast subset in SS lesions that was reduced in other conditions. Bulk RNA-Seq of a large independent patient cohort confirmed snRNA-Seq findings, including the unique and prominent interferon signature in SS. Subsequent unbiased functional experiments demonstrated that cultured primary human dermal fibroblasts—cells appreciated mainly for supporting tissue architecture—highly expressed neutrophil chemokines that bind CXCR2 following recognition of type I interferon.

Fibroblasts have historically been regarded as a homogenous cell type supporting tissue architecture; however, they are becoming appreciated as a diverse and multifunctional class of cells.^19^ Subsets of IAFs play a critical role in IL-17–mediated neutrophil recruitment during *Staphylococcus aureus* infection and psoriasis vulgaris through secretion of chemokines that bind CXCR2 and CXCR4.^18^ Unlike in the treatment of psoriasis vulgaris, biologics targeting the IL-17 pathway have not demonstrated efficacy in treating SS.^22^ Therefore, we hypothesized that IAFs promote neutrophil recruitment in SS through an IL-17–independent mechanism. We now present evidence that human dermal IAFs play a role in neutrophil recruitment in SS through recognition of type I IFN.

A 2022 study identified type I interferon-activated dermal fibroblasts in the autoimmune skin disease cutaneous lupus erythematosus using scRNA-Seq.^23^ Although type I interferon and neutrophils are well known to drive lupus pathogenesis,^24^ the connection between type I interferon and neutrophil recruitment had not been established previously. In light of our study—the first to present data supporting a role for type I interferon-activated dermal fibroblast subsets in neutrophil inflammation—these findings suggest that IAF recognition of type I interferon may contribute to neutrophil inflammation in other autoimmune diseases and interferonopathies.

Psoriasis vulgaris skin cell atlas studies have resolved IAF subsets expressing neutrophil chemokines that bind CXCR2 and CXCR4.^18,25^ Here, using a combination of subcellular resolution spatial transcriptomics and immunostaining, we identified 2 distinct neutrophil chemokine-expressing fibroblast populations: a neutrophil-proximal subset expressing CXCR2 ligands in the upper dermis and a neutrophil-distal subset expressing the CXCR4 ligand CXCL12 in the lower dermis.

These findings suggest that upper and lower dermal fibroblasts are capable of adopting a neutrophil-recruiting IAF state depending on the disease context. We speculate that CXCL12-positive fibroblasts in the lower dermis are important for initial neutrophil infiltration into the skin and that fibroblasts located in the upper dermis express CXCL1-8 to further recruit cells to the papillary dermis, where neutrophils commonly reside in patients with SS.

This current study also identified subsets of interferon-activated keratinocytes, myeloid cells, and lymphocytes enriched in SS. Similar subpopulations have been reported in cutaneous lupus erythematosus,^23^ hidradenitis suppurativa,^26,27^ and psoriasis.^25^ Future research should focus on understanding how interferon action on these cell types contributes to SS pathogenesis.

Given the diversity of conditions associated with SS, some speculate that it may lack a common underlying molecular mechanism.^1^ However, type I interferon has been implicated in these associated conditions, including inflammatory disease, infection, vaccination, pregnancy, and cancer. Furthermore, several case studies have documented SS induced by interferon therapy.^28–30^ Thus, type I interferon may serve as a unifying factor in SS, suggesting that US Food and Drug Administration–approved Janus kinase–signal transducer and activator of transcription (JAK/STAT) inhibitors, which block type I interferon signaling, could be a new therapeutic strategy. Indeed, a 2020 case study reported significant improvement of SS with baricitinib treatment.^31^

Current models of SS pathogenesis—developed from limited laboratory studies of serum and skin inflammatory mediators and case reports of targeted therapeutic trials—are incomplete but suggest that infection, cancer, and drug reactions drive heightened levels of TNFα, IL-1, and G-CSF that promote leukocytosis.^3^ In the context of hematologic cancer, aberrant malignant neutrophils further contribute to leukocytosis following treatment with G-CSF, all-trans retinoic acid, or FL3 inhibitor. T_H_1, T_H_2, and/or T_H_17 cells are thought to then promote IL-17–, TNF-α–, and/or IL-1–dependent neutrophil recruitment, activation, and extracellular trap formation.^1^ Genetic variants may increase the risk of SS development independent of malignancy. For example, a 2023 case study of a patient with cancer independent SS identified a gain-of-function *PIK3R1* mutation—specifically in neutrophils—that increased neutrophil migration and respiratory burst capacity.^2^

Consistent with current models, we observed increased expression of proinflammatory cytokines, neutrophil growth factors, and neutrophil chemokines in SS skin versus healthy skin, including IL-1, TNF-α, G-CSF (encoded by *CSF3*), and CXCR2 ligands. Contrary to the current model, but consistent with a recent case report,^22^ our data do not support a role for IL-17 in SS pathogenesis.

On the basis of our findings of increased pDCs, a prominent and unique interferon signature, type I interferon activation of dermal fibroblasts to express neutrophil chemokines, and upper dermal localization of fibroblast subsets expressing CXCR2-binding neutrophil chemokines, we speculate that inciting factors drive the production of type I interferon, IL-1, and/or TNF-α in SS. These proinflammatory cytokines then promote subsets of dermal fibroblasts to recruit neutrophils to infiltrate the skin and release extracellular traps, which in turn activate pDCs to secrete type I interferon, creating a self-sustaining inflammatory positive feedback loop.

This study has limitations that warrant discussion. First, the biopsy samples were obtained in a deidentified manner from diagnostic cases and therefore lack associated clinical data, thus precluding the use of clinical information to better understand disease heterogeneity. Second, the 1000-probe subcellular resolution spatial transcriptomics panel used here was performed on samples from patients with SS (n = 5) and a small number of HCs (n = 2) but not on samples from patients with PG. It was also not sufficient to confidently locate some cellular subsets identified by snRNA-Seq, including pDCs. Spatial transcriptomics is a rapidly evolving field, and the latest technology will resolve additional genes that may further inform future studies. Lastly, single-cell RNA-Seq has difficulty detecting neutrophils owing to factors such as fragility, high level of RNases, and low transcript abundance. The single-nucleus approach with FFPE used here has additional limitations, as fixation degrades some RNA and recovered nuclei contain a fraction of total transcript, precluding identification of a bona fide neutrophil population. Future studies using proteomics or single-cell RNA sequencing optimized for neutrophil detection will be critical for in depth characterization of neutrophils in SS and other neutrophilic dermatoses.

In summary, we have used cutting-edge single-nucleus, bulk, and spatial transcriptomics on archival clinical samples to illuminate the pathogenesis of rare human neutrophilic dermatoses. These approaches led to the identification of a unique interferon signature in SS, which was seen prominently in a subset of upper dermal fibroblasts that highly express neutrophil chemokines following recognition of type I interferon. This work provides insight into the mechanisms underlying the clinical observation of recombinant type I interferon-induced SS and identifies type I interferon and interferon-activated fibroblasts as novel therapeutic targets for the treatment of SS. Finally, the comprehensive cell atlas generated here is anticipated to be a valuable resource for the research community to facilitate future discoveries.

## Supplementary Material

1

## Clinical implications:

A strong interferon signature distinguishes SS from another neutrophilic dermatosis, namely, PG. Therapeutic interventions that block interferon signaling may represent an effective treatment for SS.

## Abbreviations used

DC: Dendritic cell
FFPE: Formalin-fixed, paraffin-embedded
HC: Healthy control
IAF: Immune-acting fibroblast
IRF1: Interferon response factor 1
pDC: Plasmacytoid dendritic cell
PG: Pyoderma gangrenosum
qPCR: Quantitative PCR
RNA-Seq: RNA sequencing
snRNA-Seq: Single-nucleus RNA sequencing
SS: Sweet syndrome
