# Supplementary material for: Positionally distinct interferon-stimulated dermal immune-acting fibroblasts promote neutrophil recruitment in Sweet syndrome
Source: J Allergy Clin Immunol. Author manuscript; Available in PMC 2025 Dec 4. (PMC12678022; doi:10.1016/j.jaci.2025.05.029)
Supplement: 1 [file NIHMS2123703-supplement-1.pdf]

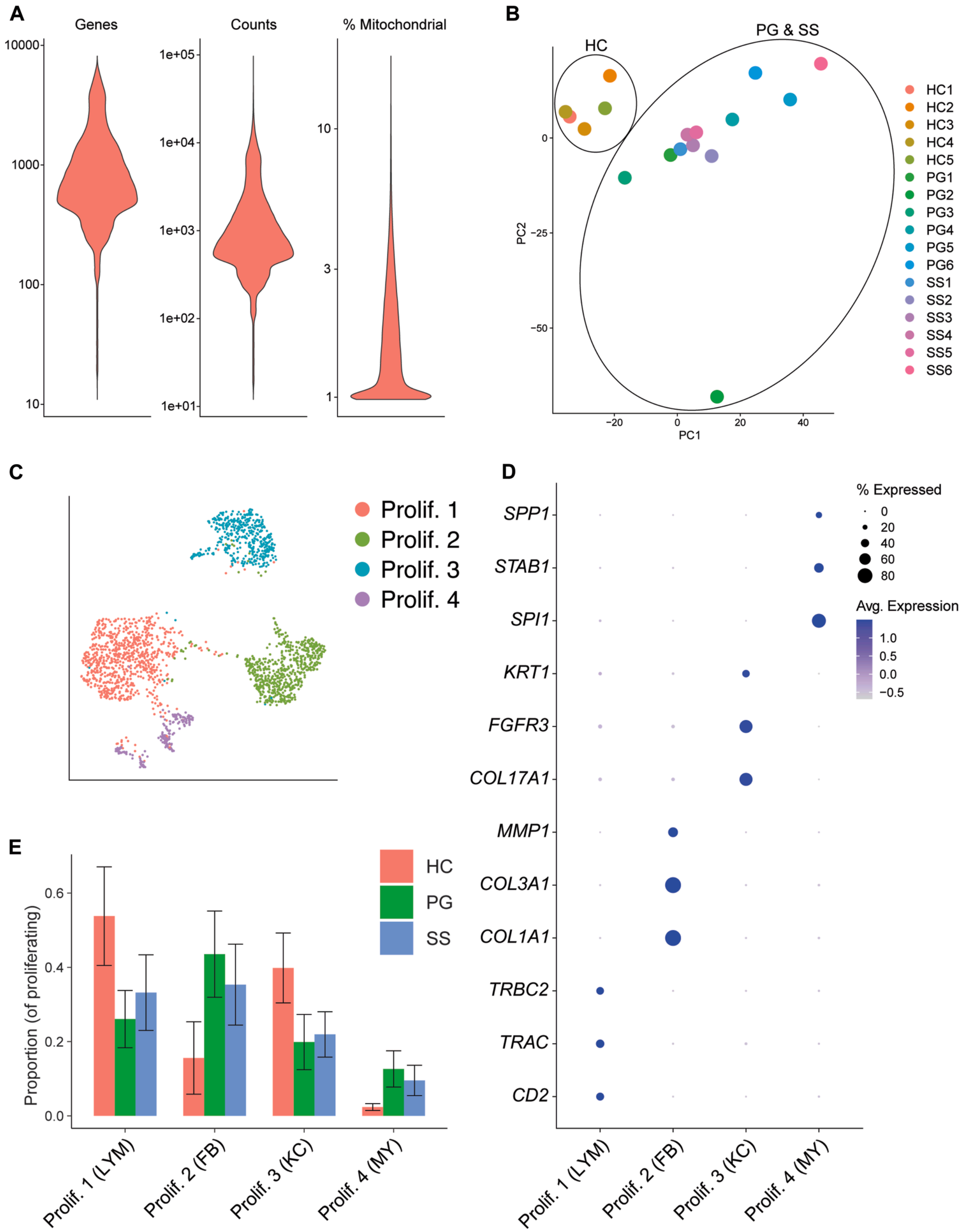

FIG E1. Single-nucleus RNA-Seq quality control and proliferating cell identification. **A,** Distribution of genes, unique counts, and percent mitochondrial reads in the snRNA-Seq data set following removal of low-quality cells and doublets. **B,** Principal component analysis of pseudobulked snRNA-Seq data. **C,** Dimensionality reduction and unsupervised clustering of proliferating cells, colored by cluster. **D,** Expression of top 3 marker genes for proliferating cell subsets. **E,** Proportion of proliferating cell subsets for each condition. Error bars represent SEMs. *FB*, Fibroblast; *IFN*, interferon; *LYM*, lymphoid cell; *KC*, keratinocyte; *MY*, myeloid cell. *Prolif.*, proliferating.

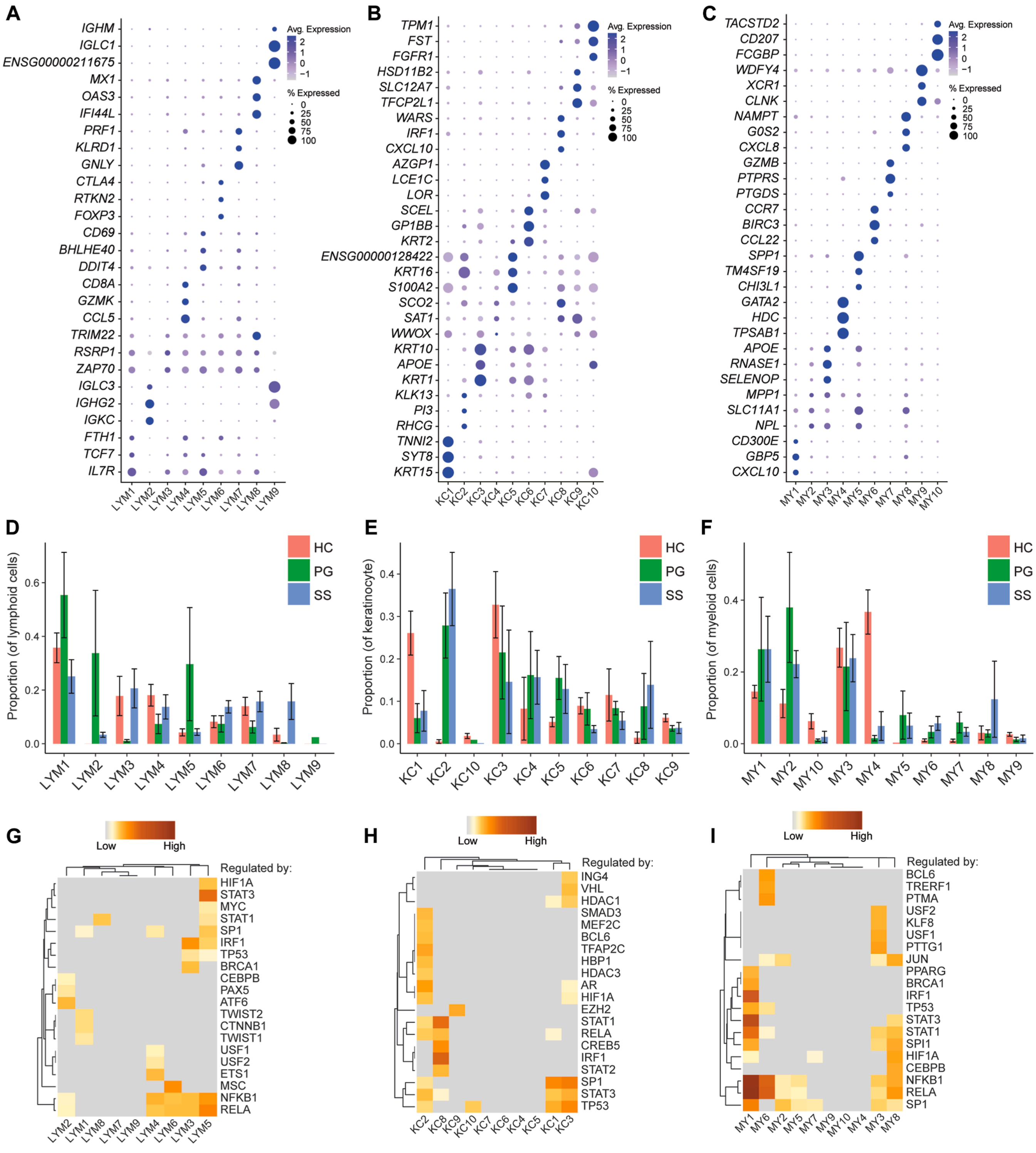

FIG E2. Interferon (IFN)-activated lymphocytes (LYMs), keratinocytes (KCs), and myeloid cells (MYs) are enriched in SS. **A**, Expression of top 3 marker genes for ILYM subsets. **B**, Expression of top 3 marker genes for KC subsets. **C**, Expression of top 3 marker genes for MY subsets. **D**, Proportion of lymphoid cell subsets for each condition. **E**, Proportion of KC subsets for each condition. **F**, Proportion of MY subsets for each condition. **G-I,** LYM (**G**), KC (**H**), and MY (**I**) subset pathway analysis using significant differentially expressed genes (P_{adj} < .05) between clusters. Error bars represent SEMs.

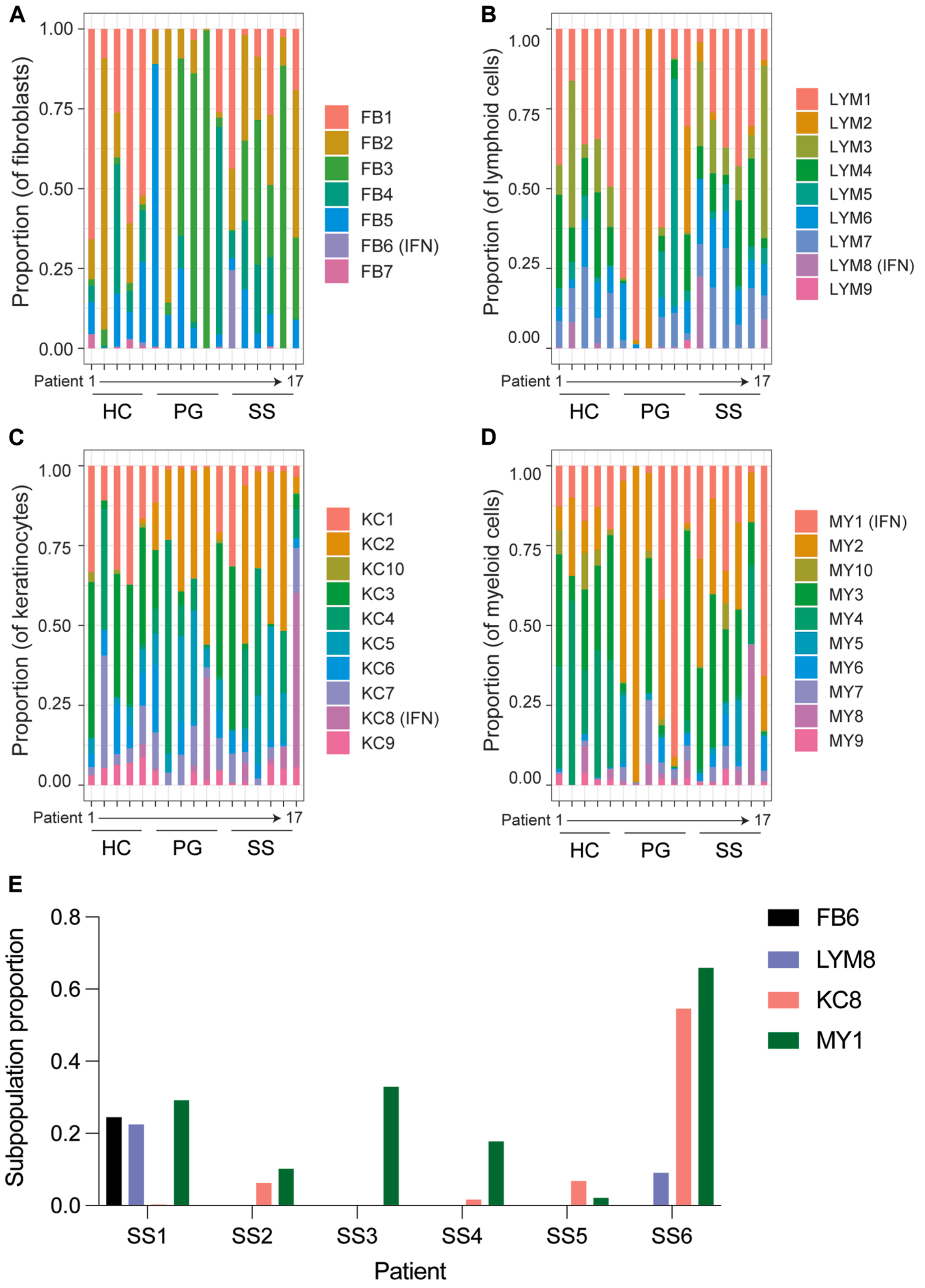

FIG E3. Cellular compartment with interferon (IFN) signature varies between patients with SS. **A,** Proportion of fibroblast (FB) subsets per patient. **B,** Proportion of lymphoid cell (LYM) subsets per patient. **C,** Proportion of keratinocyte (KC) subsets per patient. **D,** Proportion of myeloid cell (MY) subsets per patient. **E,** Proportion of IFN-activated subpopulations (of parent population) split by individual patient with SS.

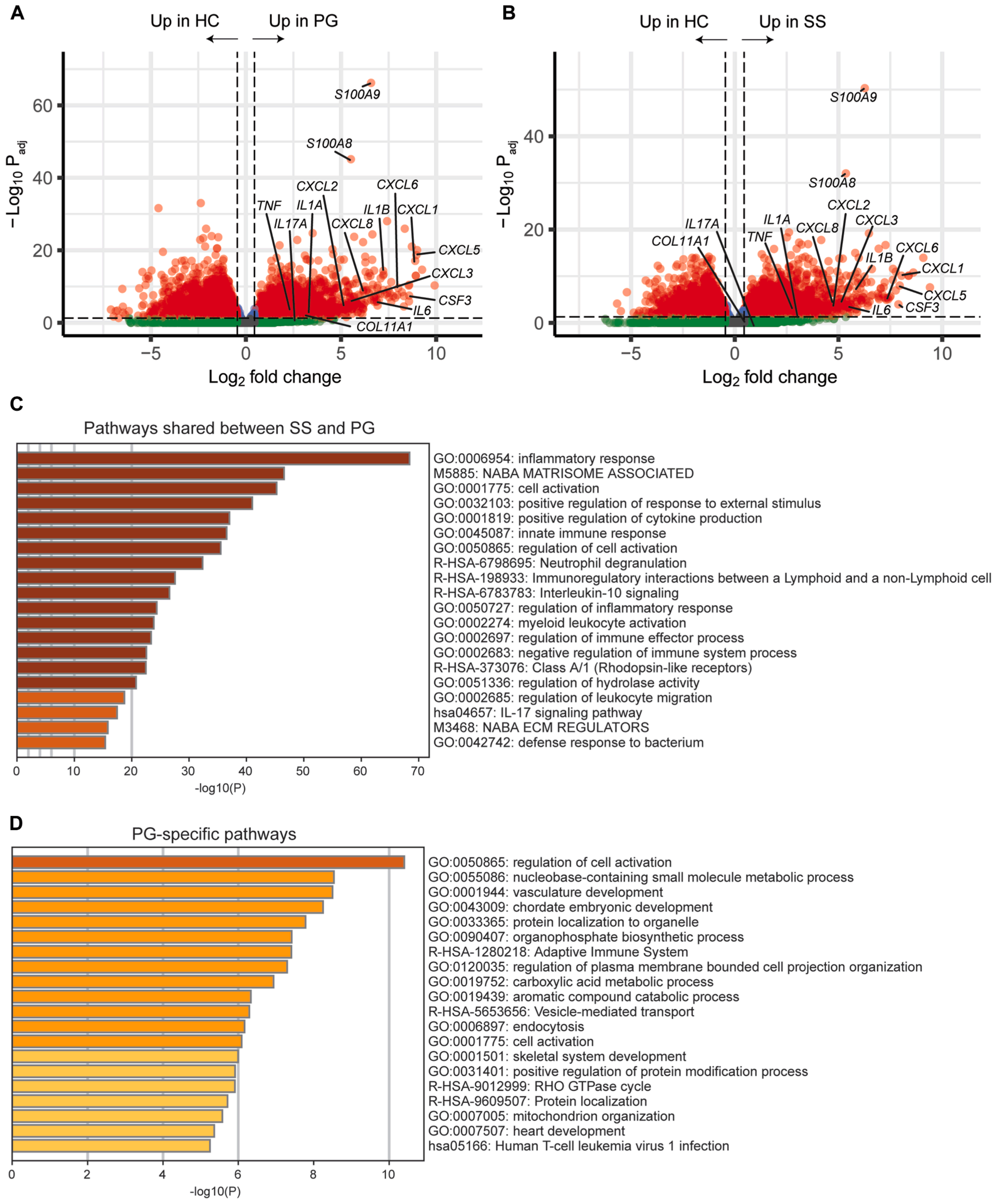

FIG E4. SS and PG bulk RNA-Seq. **A**, Differentially expressed genes in patients with PG versus in HCs. **B**, Differentially expressed genes in patients with SS versus in HCs. **A** and **B**, The adjusted *P* value (P_{adj}) threshold is .05 and $\log_2$ fold change (FC) threshold is 0.45. **C,** Pathway analysis using significantly differentially expressed genes (up and down; P_{adj} <.05) shared between patients with SS and patients with PG. **D**, Pathway analysis with differentially expressed genes unique to PG (up and down; P_{adj} < .05; |FC| > 2).

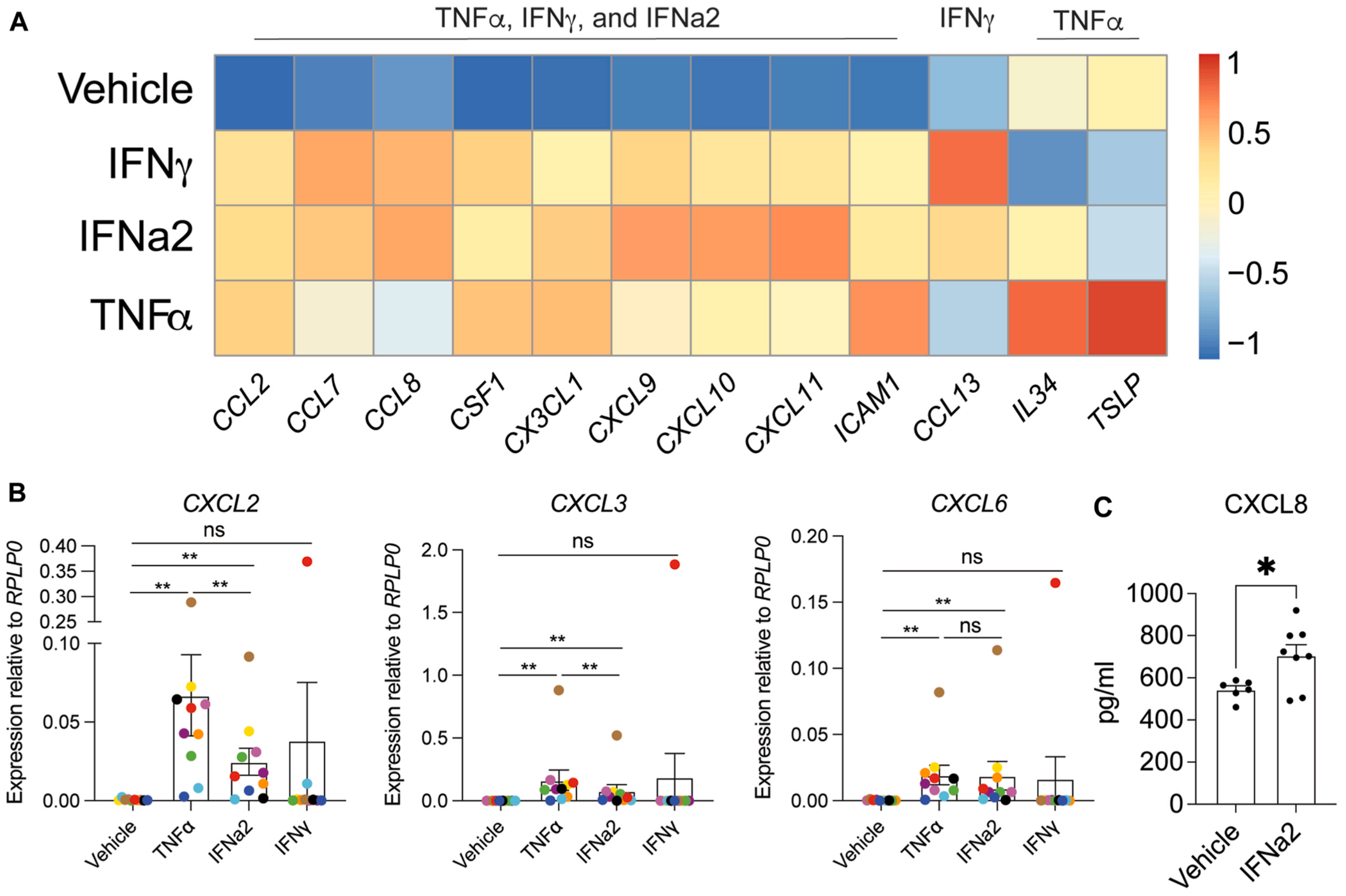

FIG E5. Bulk RNA-Seq of cultured primary human dermal fibroblasts. **A**, Select inflammatory mediator genes highly upregulated by TNF-α, IFN-γ, and IFNa2; by IFN-γ alone; or by TNF-α alone. **B**, qPCR gene expression of neutrophil chemokines. Each color represents a single donor. $^{**}P < .01$ using Wilcoxon matched pairs signed rank test. **C**, CXCL8 protein secretion measured by ELISA. $^{*}P < .05$ using an unpaired *t* test. Error bars represent SEMs. *Ns*, Not significant.

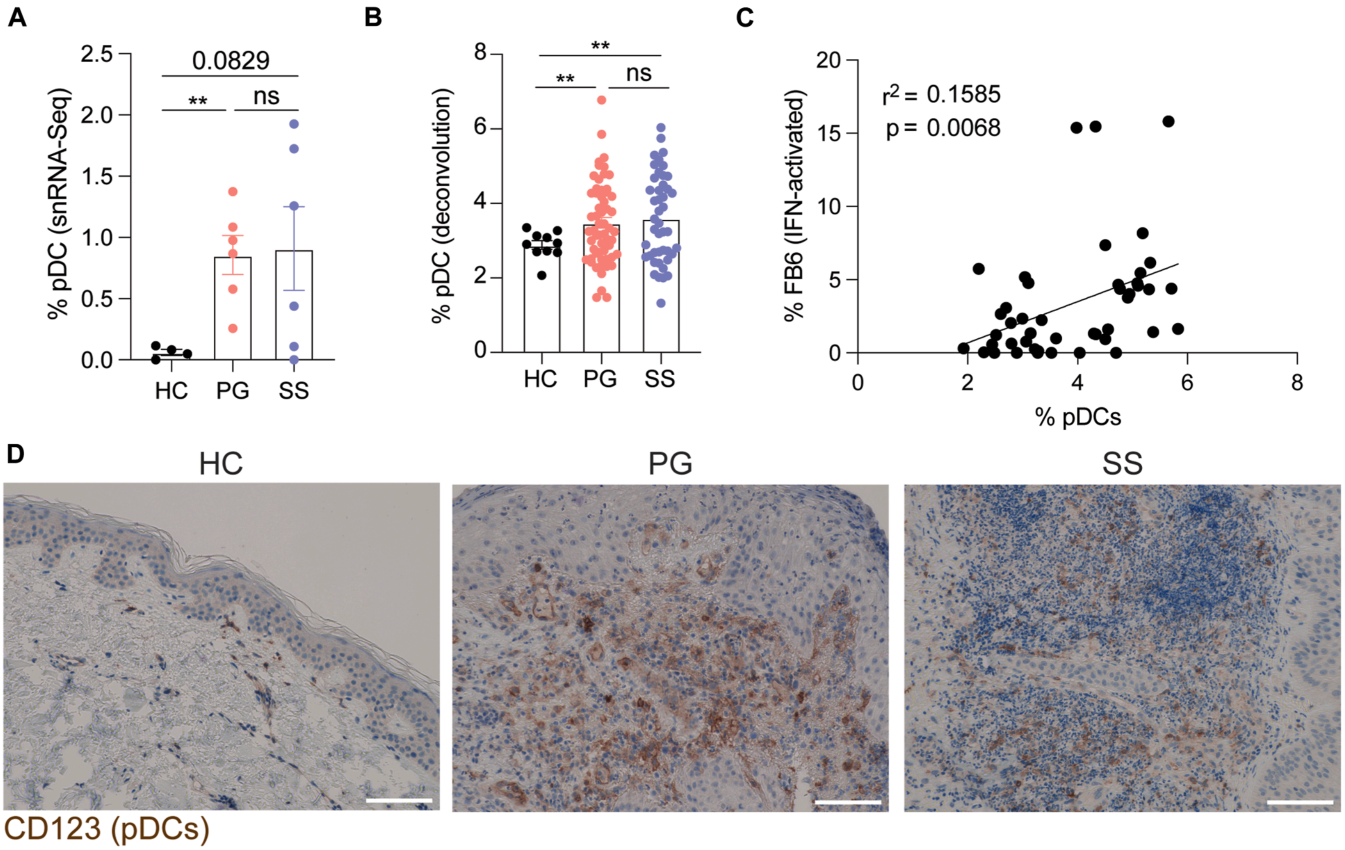

FIG E6. pDCs are enriched in neutrophilic dermatoses. **A,** snRNA-Seq quantification of pDC frequency of total cells. **B,** Machine learning–based deconvolution of bulk RNA-Seq using CIBERSORTx showing pDC frequency of total cells. **C,** Correlation between interferon-activated FB6 frequency and pDC frequency as determined by bulk RNA-Seq deconvolution. **D,** Representative CD123 staining that marks pDCs in HCs, patients with PG, and patients with SS. The experiment was conducted twice. Scale bar = 150 μm. $^{**}P < .01$ using unpaired *t* tests. Error bars represent SEMs. *ns,* Not significant.

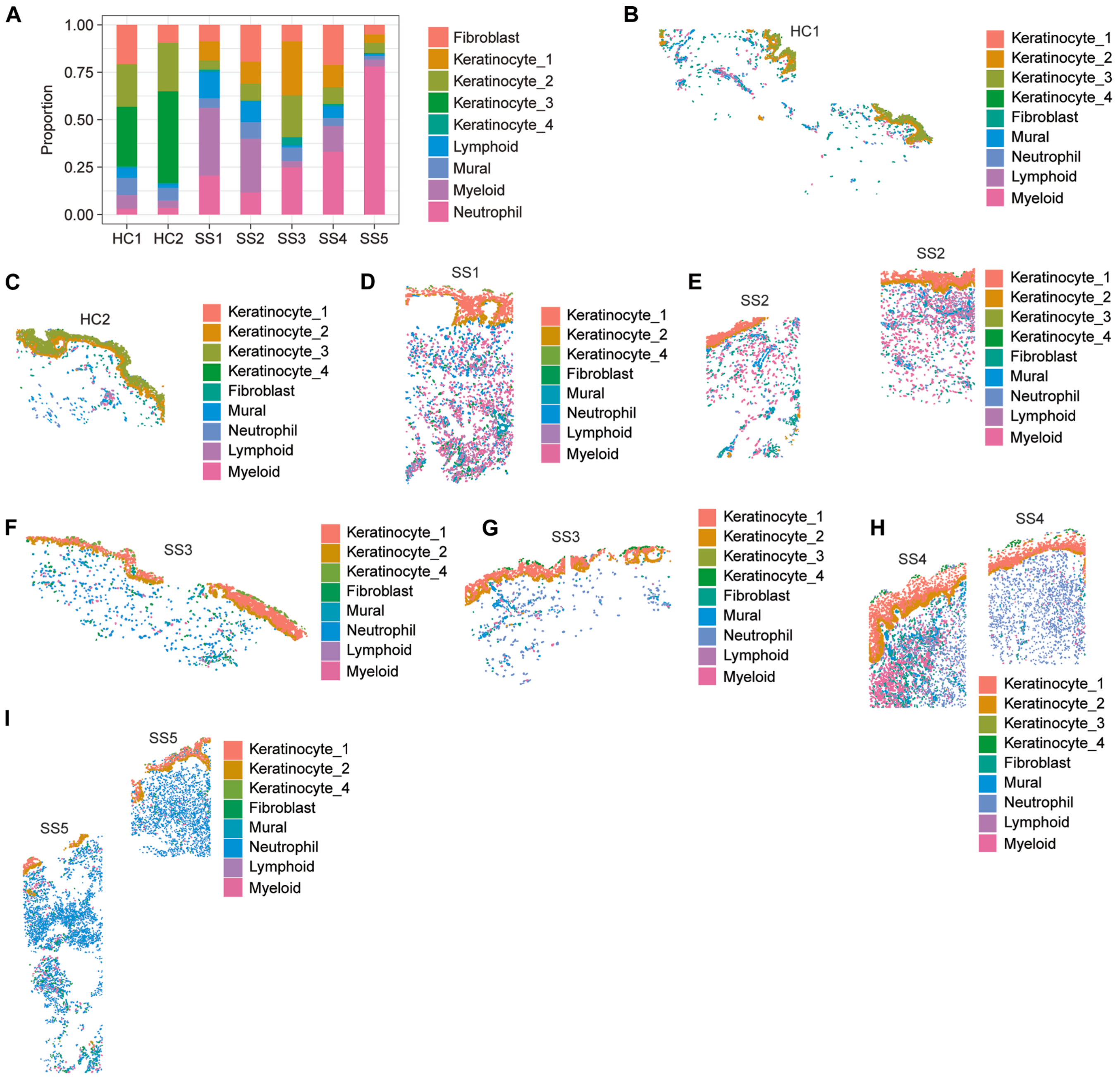

FIG E7. Subcellular resolution spatial transcriptomics of SS. **A,** Proportion of each cluster across patients. **B-I,** Clusters projected onto tissue sections for each patient.

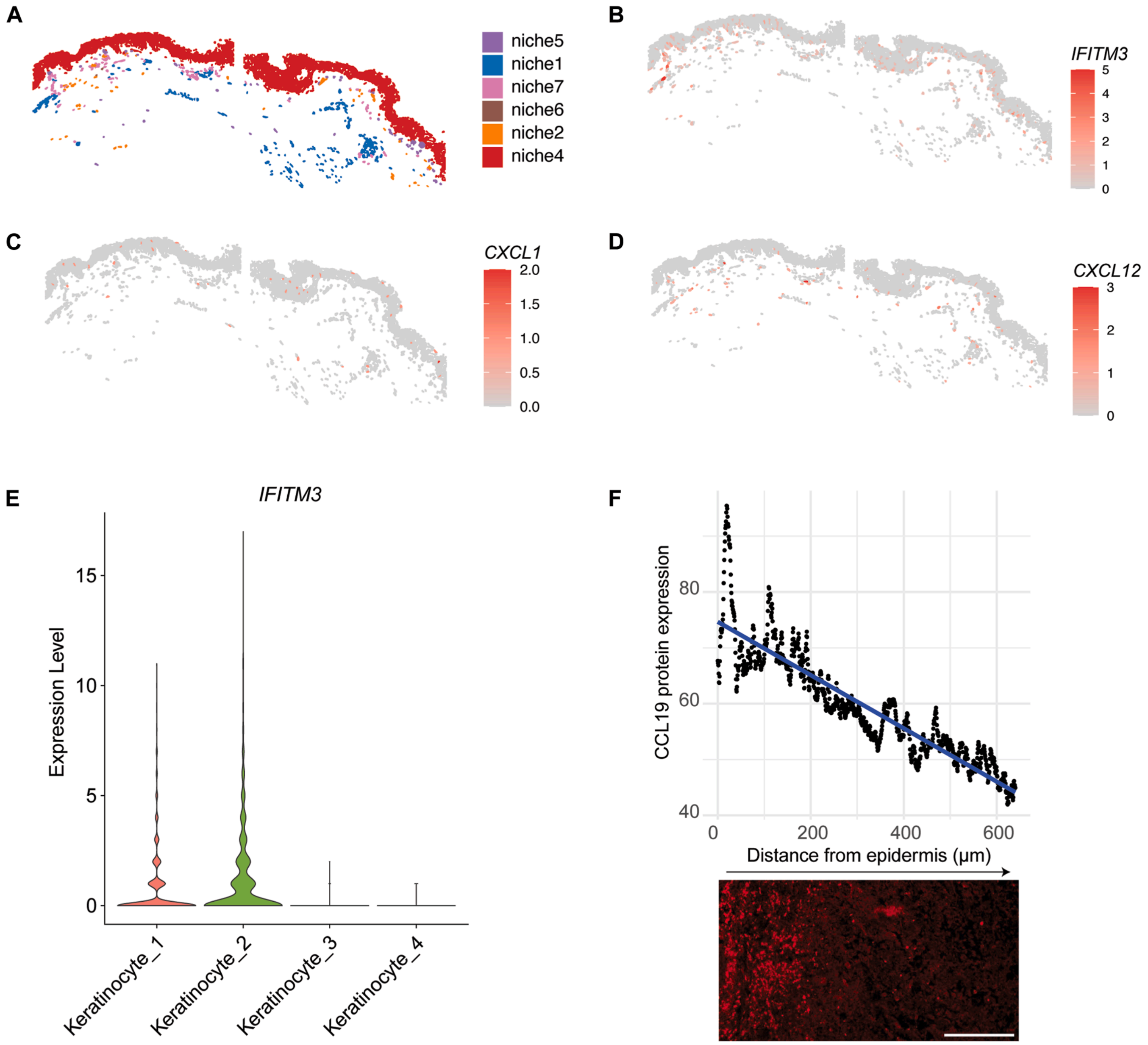

FIG E8. Spatial transcriptomic niche analysis of SS. **A,** Niche analysis projected onto HC tissue. **B-E,** Expression of interferon-induced gene *IFITM3* (**B**), *CXCL1* (**C**), and *CXCL12* (**D**) in HCs. **E,** Expression of *IFITM3* across keratinocyte subsets (includes both conditions). **F,** Representative protein immunostaining of CCL19 in SS (*bottom*) and quantification of expression as a function of distance from epidermis (*top*). The experiment was conducted twice. Scale bar = 150 μm.
